# Biomechanical Modulation Therapy—A Stem Cell Therapy Without Stem Cells for the Treatment of Severe Ocular Burns

**DOI:** 10.1167/tvst.9.12.5

**Published:** 2020-11-02

**Authors:** Ricardo M. Gouveia, Che J. Connon

**Affiliations:** 1 Biosciences Institute, Faculty of Medical Sciences, The Medical School, Newcastle University, Newcastle upon Tyne, UK

**Keywords:** corneal biomechanics, corneal burns, limbal epithelial stem cells, cell-based therapy, regeneration

## Abstract

Ocular injuries caused by chemical and thermal burns are often unmanageable and frequently result in disfigurement, corneal haze/opacification, and vision loss. Currently, a considerable number of surgical and pharmacological approaches are available to treat such injuries at either an acute or a chronic stage. However, these existing interventions are mainly directed at (and limited to) suppressing corneal inflammation and neovascularization while promoting re-epithelialization. Reconstruction of the ocular surface represents a suitable but last-option recourse in cases where epithelial healing is severely impaired, such as due to limbal stem cell deficiency. In this concise review, we discuss how biomechanical modulation therapy (BMT) may represent a more effective approach to promoting the regeneration of ocular tissues affected by burn injuries via restoration of the limbal stem cell niche. Specifically, the scientific basis supporting this new therapeutic modality is described, along with our growing understanding of the role that tissue biomechanics plays in stem cell fate and function. The potential impact of BMT as a future treatment option for the management of injuries affecting tissue compliance is also further discussed.

## Introduction

Burns are among the most frequently reported causes of eye injuries and are estimated to account up to 18% of all ocular traumas, most prominently in younger men and children.^1^ The injuries caused by chemical and thermal burns to the eye can range from mild unilateral conjunctival or corneal epithelial damage to sight-threatening damage to the conjunctiva and cornea.^2^ Together, unilateral and bilateral corneal blindness is estimated to affect up to 28 million people worldwide.^3^ The resulting vision impairment and blindness have important and life-long health, socioeconomic, and quality-of-life implications for individuals and represent a substantial impact to healthcare systems, with yearly global economic costs estimated between $3 and $42 billion.^4^^–^^6^ In this context, there is a pressing need for new, affordable, and accessible therapies aimed at either preventing or restoring burn-induced vision loss. Historically, the development of clinical solutions for these conditions has relied heavily on repurposed treatments (e.g., from the dermatology field), as well as on the analysis of clinical cases and subsequent identification of discrete therapeutic markers. The strategy for most existing therapies is predominantly focused on injury management and minimization of post-injury damage. More recently, advances in the field of regenerative medicine have allowed the development of new approaches aimed at injury remediation, with new stem cell transplantation methods providing a particularly effective way to restore corneal function. However, our understanding of how these better clinical outcomes are achieved at the cellular and molecular levels remains limited. Here, we explore how biomechanical modulation therapy (BMT), a new treatment modality based on recent discoveries in corneal biomechanics, is being translated from new insights on the fundaments of corneal stem cell biology to provide a regenerative solution to corneal burns. The origins of BMT are reviewed, along with a critique on how this approach compares with currently existing therapies and what to expect from future developments.

## Current Treatments for Corneal Burns

In many cases, a timely intervention is key to preventing significant functional and anatomical burn damage to the ocular structures. Typically, this involves clearing the injuring agent from the eye surface via ocular lavage (e.g., with water, saline, or neutralizing agents) followed by pressure patching and suppressing inflammation and infection at the acute phase of injury until re-epithelization occurs.^2^

Controlling the inflammatory process represents a particularly crucial aspect in the post-traumatic management of ocular burns, during both its acute and chronic phases.^7^ For example, local corticosteroids such as dexamethasone are effective in reducing inflammation by decreasing neutrophil invasion and inhibiting matrix-degrading enzymes.^8^ However, they can also reduce corneal stromal repair and delay fibrosis.^9^ Moreover, the topical and parenteral administration of antibiotics (e.g., tetracycline) or ascorbic acid has been reported to prevent corneal ulceration and to facilitate fibrosis.^10^

**Figure 1.**
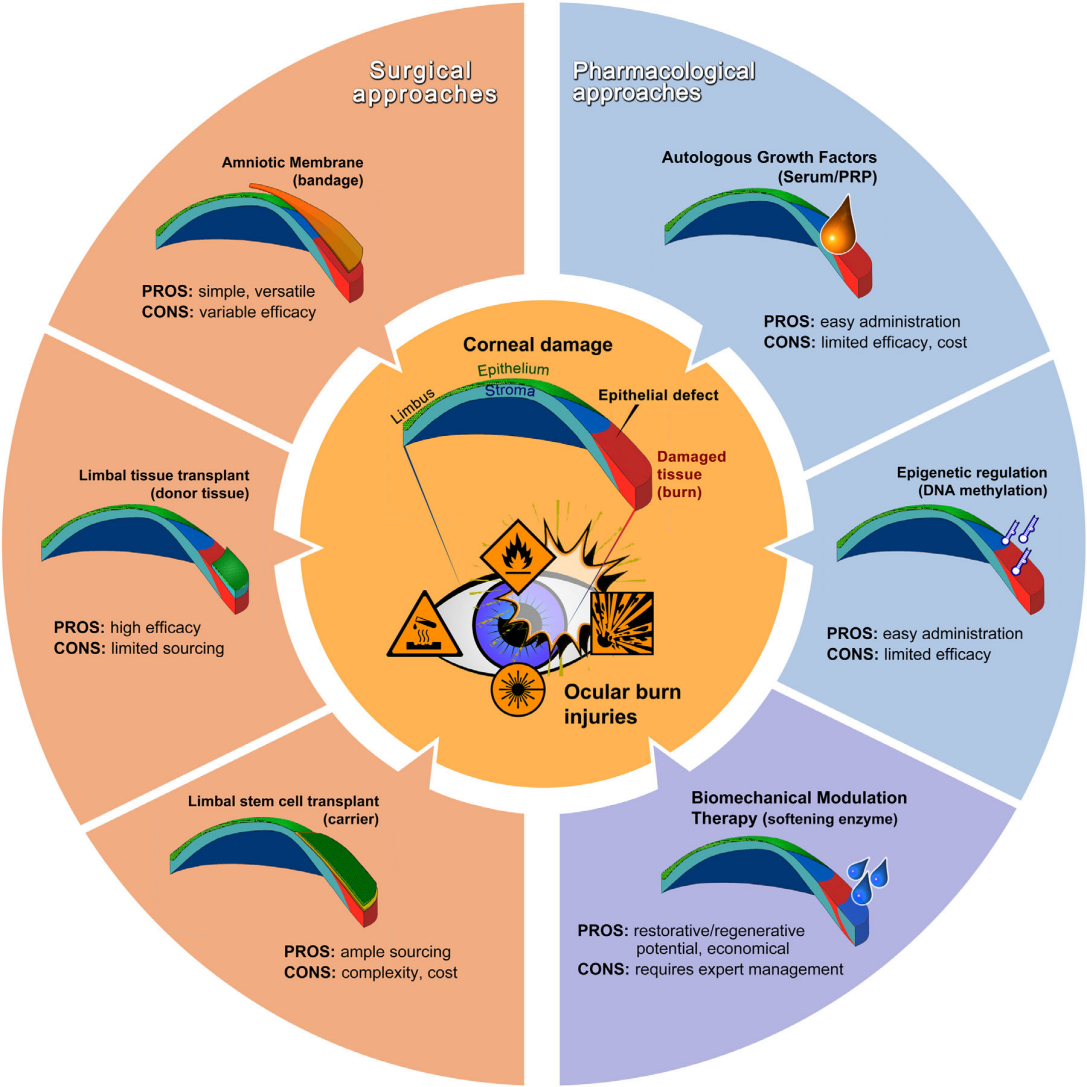
Current treatments for corneal burns and how BMT fits in their context. As the anterior part of the eye, the cornea is vulnerable to burn injuries, including those caused by chemicals, heat, explosions, and radiation. In severe cases, extensive burns can affect the limbus, an anatomical, biochemical, and biophysical niche in the periphery of the cornea where epithelial stem/progenitor cells reside. Damage to this area can subsequently compromise stem cell self-renewal and lead to chronic epithelial defects and vision loss. Available therapies can be broadly classified as surgical or pharmacological in approach, with different treatments presenting corresponding advantages and disadvantages. BMT represents the only pharmacological approach with restorative/regenerative potential; however, its safety and efficacy depend on expert intervention and may preclude outpatient administration.

In more severe cases, however, or when these frontline treatments are not readily available, ocular burns can cause a rapid and progressive destruction of the corneal surface and lead to serious anterior segment complications and blindness.^11^ Severe ocular burns are also the most common cause of limbal stem cell deficiency (LSCD) and are characterized by permanent non-healing epithelial defects, perilimbal ischemia, stromal inflammation, neovascularization, conjunctivalization and corneal haze/opacification, and edema.^12^^,^^13^ Managing such complications in chronic phases of the injury normally requires a combination of different treatments. Current and experimental treatments can generally be grouped into either surgical or pharmacological approaches (Fig. 1). Their relevance and impact are briefly overviewed, along with their main advantages and disadvantages.

## Surgical Intervention for the Treatment of Corneal Burns

Surgical treatments generally provide an effective way to restore vision after severe corneal burns, particularly when such injuries affect a large portion of the limbus (i.e., the region at the periphery of the cornea where epithelial stem cells reside).^14^ Currently, surgical procedures are comprised mostly of the application of protective bandages containing healing factors (e.g., human amniotic membrane) to accelerate corneal re-epithelialization and, in more severe cases, the replacement of limbal epithelial stem cells (LESCs) lost due to injury. The latter is achieved by transplantation, either of limbal tissue from donors or of stem cells expanded ex vivo (Fig. 1). Surgical interventions tend to be highly efficient methods to repair corneal function; however, they depend on highly trained personnel and imply higher costs associated with operating theater procedures and donor stem cell and tissue sourcing.

### Human Amniotic Membrane Bandage

The first therapeutic use of human amniotic membrane (hAM) in ophthalmic surgery was described 80 years ago, when it was used as bandage material for the management of conjunctival defects.^15^ Since then, hAM has become a common surgical adjunct for the treatment of many other eye conditions, including chemical and thermal burns in the cornea,^16^ as extensively reviewed recently.^17^ Despite the clinical efficacy of hAM in multiple forms (fresh, dried, cryopreserved) and its widespread applications (as permanent graft or temporary patch at acute phases),^18^ its therapeutic efficacy is variable, and its mechanism of action remains mostly unexplained (Fig. 1). The hAM is a thin, translucent, and sturdy tissue comprised of a single-layer epithelium, a thick basement membrane, and an avascular stroma enriched in anti-angiogenic, anti-inflammatory, and anti-scarring growth factors.^19^ These growth factors, therefore, have been associated with the ability of hAM to stimulate wound healing (i.e., by promoting re-epithelialization, controlling inflammation, and preventing scarring),^20^ but evidence of this effect is still limited and requires further scientific substantiation.^21^ Moreover, the use of hAM for treating severe eye burns is limited and restricted to cases where a substantial population of LESCs remains viable. When wider LESC deficiency ensues (e.g., occurrence of extensive limbal blanching), then hAM transplantation alone is not sufficient for promoting an effective regeneration of the cornea, and the additional transplantation of autologous or heterologous stem cells is required.

### Limbal Tissue Transplantation

In severe burn injuries leading to extensive LSCD, recreation of a suitable environment for hosting a functioning new population of LESCs typically first requires chronic phase conditions where ocular surface inflammation has subsided or is controlled with medication.^22^ Management methods involving transplantation of limbal tissue from the healthy fellow eye are considered the most effective surgical procedure for replacing the affected limbus in patients with total unilateral LSCD. Examples include conjunctival–limbal autograft^23^ and simple limbal epithelial transplantation, in which a 2 × 2-mm strip of donor limbal tissue from the healthy eye is divided into 8 to 10 small pieces and is evenly distributed over an amniotic membrane placed on the cornea.^24^ In patients with bilateral LSCD, living-related conjunctival–limbal allografts from immediate family members^25^ and keratolimbal allografts from cadaveric donors^26^^,^^27^ are also effective surgical alternatives. These techniques have the advantage of being slightly less limited by tissue availability (Fig. 1); however, complications primarily arise from immunologic rejection, chronic ocular surface exposure, and graft-related complications (thickness, position, and alignment).^28^^,^^29^

### Limbal Stem Cell Transplantation

Alternatively, surgery can be used to reconstruct the limbus niche by transplanting LESCs from autologous or allogeneic sources on carriers (Fig. 1), typically hAM,^30^ fibrin,^31^ or collagen-based hydrogels.^32^ A number of techniques involving cultivated limbal epithelial transplantation exist, for both total unilateral LSCD (using cell biopsies from the contralateral eye)^33^ and bilateral LSCD (using cells isolated from donor corneas).^34^ Additional surgical options for bilateral LSCD using autologous stem cells include cultivated oral mucosal epithelial transplantation^35^ and the transplantation of autologous conjunctival epithelial cells cultivated ex vivo on denuded hAM grafts.^36^ All of these techniques share the advantage of a wider availability of transplantable stem cells; however, they are limited by the complexity and cost inherent to the ex vivo cultivation process (Fig. 1). Moreover, similar to approaches involving limbal tissue transplantation, the success of these interventions requires inflammation suppression and preparation of the host tissue, with careful removal of its necrotic areas and further tissue reconstruction, such as by advancing viable Tenon's layer (tenonplasty), lamellar keratoplasty, or deep anterior lamellar keratoplasty for patients with extensive stromal scarring.^11^ A more extensive review of these practices and outcomes can be found in a recent report from the European Vision Institute.^37^

### Pharmacological Treatment of Corneal Burns

Using pharmacological approaches to treat corneal burn injuries has the advantage of being considerably less demanding in terms of technical complexity, material sourcing, process administration, and costs. However, the effectiveness of these approaches depends greatly on a robust understanding of corneal cell and stem cell biology, as well as of the mechanisms regulating tissue healing and homeostasis. Acquiring such knowledge has been the focus of decades of research, and will arguably lead to more targeted and sophisticated therapeutic solutions based on strong empirical evidence. Currently, pharmacological treatments for severe ocular burns are few and limited in both scope and efficacy but nevertheless remain attractive alternatives or adjunct solutions to other approaches, as indicated in a recent and excellent analysis on this subject.^38^

### Autologous Growth Factors

Human serum contains many soluble factors shown to promote healing in many tissue types, including the cornea.^39^ Topical applications of autologous serum isolated from whole blood^40^ or from the umbilical cord^41^ have been shown to be effective in promoting wound healing in patients with persistent epithelial defects and in accelerating epithelial healing in acute chemical injuries. In addition, variation of autologous serum comprising platelet-rich plasma (PRP) has been tested topically^42^ and via subconjunctival injection in patients with ocular chemical injuries.^43^ The mechanism of action of PRP is likely the same as that of autologous serum but more effective in promoting epithelial healing due to its higher concentration in growth factors.^44^ Furthermore, fibrinogen-depleted human platelet lysate is currently undergoing clinical trials for graft-versus-host disease in the United States. However, the complexity and costs associated with isolating serum and PRP,^45^ along with their inability to promote the restoration of the limbal stem cell niche, represent important limitations of this treatments (Fig. 1). As such, and despite their safety and easy administration, these sera will mostly be used as adjuncts to standard medical treatments and only until fully synthetic equivalents are developed.

### Epigenetic Regulation

More recently, advances in understanding the role of cellular epigenetics in eye development and function have resulted in the first steps to translate epigenetic regulation (e.g., via control of DNA methylation, histone and nonhistone posttranslational modifications, and non-coding RNA regulators) into new clinical and ophthalmological applications. For example, hypermethylation of lysine 4 and hypomethylation of lysine 27 on the histone H3 protein at the *TGFBIp* locus are putative pathogenic mechanisms involved in corneal dystrophies, including ocular surface fibrosis and impaired wound healing.^46^^,^^47^ DNA methyltransferates (DNMTs) have also been shown to play a role during corneal epithelial wound healing, with increased expression of DNMT1 and DNMT3B contributing to the control of epithelial cell migration, differentiation, and proliferation.^48^ Increasing evidence thus suggests that epigenetic regulators represent promising targets for controlling corneal stem cell behavior and promoting corneal epithelial healing post-injury through the use of highly selective and easily deliverable molecular biology tools (Fig. 1). However, corneal burn treatments based on epigenetic regulation will probably have limitations similar to those relying on growth factors, with little or no impact on the overall restoration of the damaged limbus niche.

## Overall Limitations of Current Treatments

At present, no single treatment has been developed to address all possible corneal burn injuries, as these vary greatly in terms of nature of the offending agent, the extension and severity of damage, the patient's particular healing response, and the availability of resources. In practice, decisions on the best therapeutic approach to treat corneal burns are made on a case-by-case basis, with one or more treatments being performed, often iteratively and usually involving both surgical and pharmacological methods, to repair the damage to the LESC population of the cornea, promote re-epithelialization, and restore homeostasis. We propose that, in part, this complex ad hoc approach derives from an insufficient understanding of how burns can temporarily or even permanently change the biophysical and biochemical properties of corneal tissues and how these sequelae subsequently affect the specific molecular pathways involved in corneal repair at a cellular level. Conversely, it is reasonable to suggest that uncovering the fundamental mechanisms of corneal development and function will help us devise more universal, accessible, and effective treatments able to recruit the inherent processes of tissue healing and regeneration.

## Corneal Biomechanics in Health and Disease

The role of tissue biomechanics in corneal function, and in particular as a regulator of LESC behavior, has been the focus of growing research in the past decade.^49^ Studies using contact^50^ and non-contact^51^ analytical tools have demonstrated that the corneal limbus represents a biomechanical niche distinct from its central region. Specifically, the high-resolution characterization of corneal biomechanics shows that the matrix supporting epithelial cells in the limbus is significantly more heterogeneous compared with that supporting the central epithelium, and is comprised of numerous pockets with significantly lower elastic modulus associated with LESC residency.^52^ This inherent compliance of the limbus has also been shown to be sensed by and translated within LESCs via YAP-dependent mechanotransduction pathways.^53^^,^^54^ Together with its co-effector TAZ, YAP is a well-known molecular regulator of stem cell fate^55^^,^^56^ and is crucially involved in downstream signaling promoting LESC maintenance, proliferation, and stratification.^57^^,^^58^ Moreover, YAP inactivation in response to limbus-like matrix compliance has been associated with both direct and indirect upregulation of the LESC markers ΔNp63, Wnt/β-catenin, and ABCG2.^52^^,^^58^ Conversely, the stiffening of the limbus matrix (e.g., due to burn injury or fibrosis) has been shown to rapidly change LESC phenotype in multiple species,^52^^,^^59^ with YAP activation and nuclear translocation initiating a signaling cascade leading to increased cell activation, migration, and differentiation via suppression of ΔNp63 and Wnt/β-catenin signaling and increased expression of BMP4.^58^ Numerous studies indicate that these mechanisms of action not only regulate stem cell response in the cornea and other epithelia^60^ but might also provide the link between chronic inflammation and LSCD,^61^ metaplasia,^62^ and subsequent vision loss.^63^^,^^64^

Although the course of ocular burns depends on the nature of its agent, most severe cases end up affecting the biomechanical properties of the anterior cornea, including in the limbus. This shared effect is particularly evident in alkali burns due to the lipophilic nature of many basic substances, which are thus capable of rapidly penetrating the eye and causing irreversible intraocular damage in as little as 5 minutes.^1^ Such penetrating burns result in greater epithelial disruption and destruction of the underlying proteoglycan ground substance, leading to an immediate tissue stiffening.^52^^,^^65^ Acid injuries typically cause protein coagulation and precipitation in the epithelium, forming a barrier that further limits penetration of the burning agent deeper into the eye.^66^^,^^67^ Corneal injuries caused by thermal agents (e.g., resulting from exposure to scalding liquids, direct flame, or burning items)^68^ and radiation exposure (e.g., UV light)^69^ tend to be rarer and less severe. However, and similar to chemical injuries, thermal and radiation damage to the cornea can result in further stiffening at later stages due to tissue contracture and fibrosis.^12^^,^^65^ Changes in corneal biomechanics, and in particular the stiffening of the limbus milieu, are therefore recognized as one of the most common and long-lasting, albeit less evident, consequences in all different types of burn injuries. Strikingly, and despite surgical approaches providing strong evidence that successful LESC maintenance post-burn requires the recreation of a limbus-like environment (e.g., via tenonplasty, by using limbal matrix or soft carriers in transplants), no therapy currently exists using restoration of corneal biomechanics as a direct way to improve healing outcomes.

## Biomechanical Modulation Therapy

The potential clinical application of LESC phenotype-through-biomechanical modulation is a new concept that has recently been investigated. Research has shown that the localized use of low doses of collagenase type I is a simple but efficient method to soften collagen-based substrates in vitro by reducing their density and in this way reproduce the compliance of the natural limbus.^52^^,^^58^ Moreover, LESCs grown on these softer substrates showed increased expression of LESC-characteristic markers and lower YAP expression and activation, which in turn promotes LESC maintenance, proliferation, stratification, and survival. But, most importantly, this enzymatic strategy was then successfully shown to change the mechanical stiffness both in vivo and ex vivo while maintaining overall tissue structure (i.e., without causing tissue melting or ectasia). Being dependent on enzymatic activity alone also makes it versatile, allowing both dose and duration of treatment to be changed while maintaining optimal efficiency. For example, the topical use of collagenase type I at 200 µg·mL^−1^ for 15 minutes (equivalent to 2 units·cm^–^^2^·hr^–^^1^ of total collagenase activity) to soften small areas in the anterior surface of the central cornea allowed the (re)creation of a limbus-like mechanical and phenotypic milieu in rabbits. Subsequently, these softened areas were shown to serve as niches for epithelial stem/progenitor cell maintenance in vivo. The softening treatment did not compromise the integrity of the tissue, nor did it cause any measurable inflammation or neovascularization.^52^ A similar approach was successfully taken within tissue engineering, creating a pseudo-limbus area in collagen hydrogels; however, as treatment duration is not a critical factor in vitro, collagenase was applied at a reduced concentration (50 µg·mL^−1^) for a longer time (60 minutes) to achieve the intended biomechanical outcome.^52^ Cells residing on collagenase-treated areas better retained an undifferentiated, LESC-like behavior, whereas cells on stiffer, untreated areas assumed a more differentiated phenotype.^52^^,^^53^^,^^58^ This modulation of cell fate was shown to depend on the biomechanical similarity of the substrate with the natural limbus^52^^,^^53^^,^^70^ and not on structural or compositional changes (e.g., interactions with new topographical or biochemical cues exposed by the enzyme treatment).

The therapeutic use of tissue-softening enzymes for restoring tissue biomechanics after a burn injury has also been explored. Specifically, trials performed in rabbits have demonstrated that a topical, 15-minute application of collagenase at 200 µg·mL^−1^ can fully reverse the stiffening of the corneal limbus caused by alkali damage,^52^ effectively restoring its natural capacity to support LESCs. These trials showed that the softening treatment did not compromise the integrity of the cornea, nor did it result in changes in intraocular pressure.^52^ Moreover, it prevented epithelial cells (either surviving the injury or repopulating the burn from intact areas) from becoming activated via YAP signaling, and contributed instead to LESC maintenance and epithelial recovery. Conversely, the increased stiffness of the limbus caused by alkali burns (and not subsequently treated with collagenase) induced the remaining epithelial cells to differentiate, leading to a depleted LESC population.^52^

**Figure 2.**
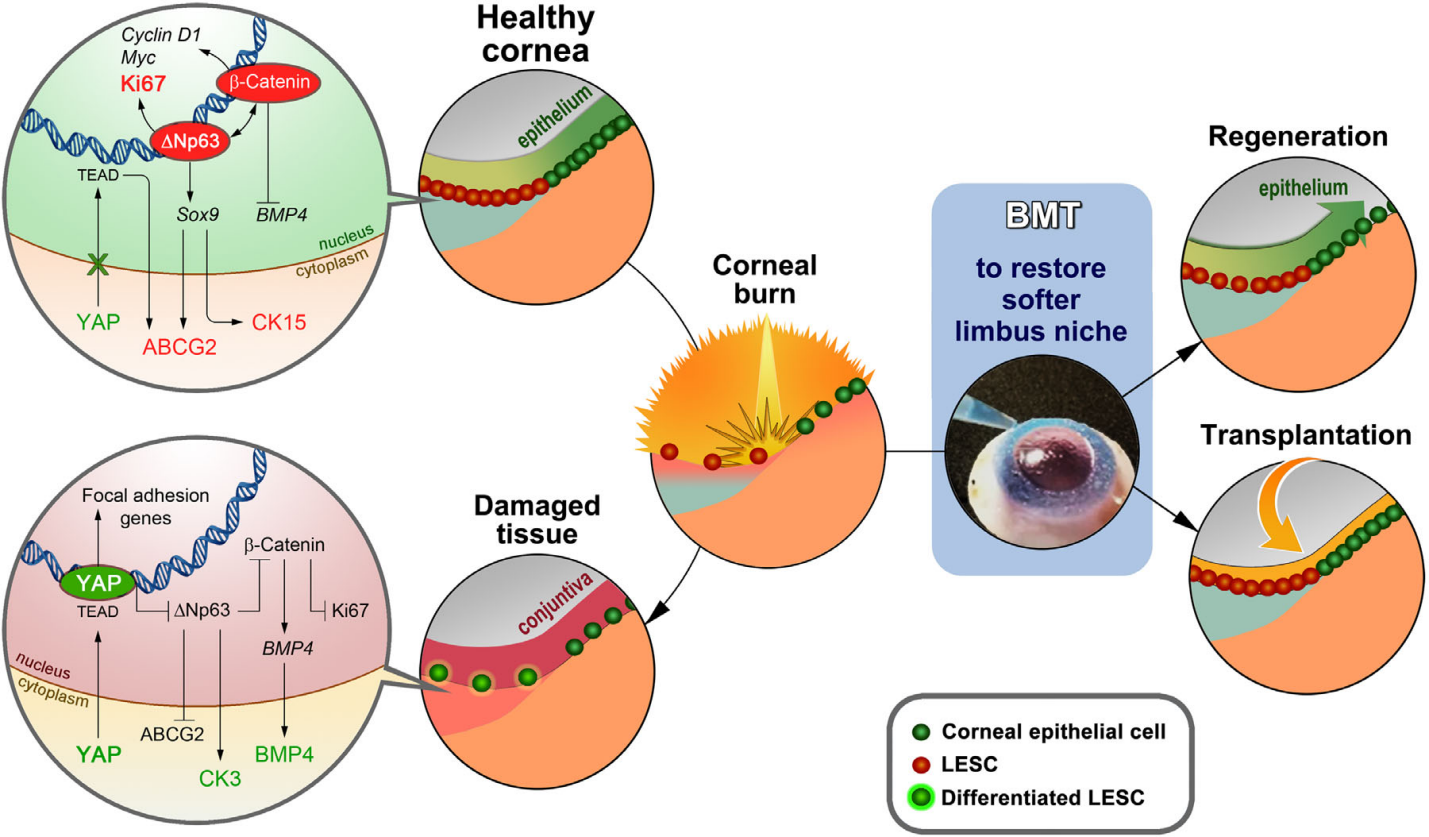
BMT and its mechanisms of action. Recent studies strongly support the notion that LESC maintenance is regulated by tissue compliance within the limbus niche, via YAP-dependent mechanotransduction pathways (*top left*). In cases where stiffening of the corneal limbus occurs (e.g., following burn injuries), LESCs are similarly affected by mechanotransduction signaling, with LESC differentiation and loss consequently leading to impaired healing, limited re-epithelialization, and conjuntivalization (*bottom left*). BMT represents a new pharmacological approach to prevent and treat such impairments (*right*). Using a low-dose, short-duration, localized application of a tissue-softening enzyme, BMT can restore the natural biomechanics of the damaged limbus. The restored limbus can thus provide a suitable substrate allowing surviving LESCs to grow, proliferate, and promote tissue regeneration (i). Alternatively, in more extensive burns where no viable LESCs remain, limbus restoration via BMT may act as an adjunct treatment for supporting stem cell expansion and residency after LESC or limbal tissue transplantation (ii).

As a drug, collagenase has already received U.S. Food and Drug Administration and European Medicines Agency approval for treating several types of conditions affecting connective tissues, namely contractures.^71^ In this perspective, the enzyme represents a safe candidate for the off-label modulation of tissue biomechanics and subsequent regulation of (stem) cell phenotype. Such a pharmacological approach, better described as biomechanical modulation therapy, or BMT, could have important and extensive applications, namely as a non-hormonal therapeutic method to regulate stem cell fate and function via softening of pathologically hardened tissues (Fig. 2). Modulation of tissue biomechanics is not, however, restricted to the use of proteolytic enzymes, as feasible alternatives include intra-tissue injection of natural and/or synthetic proteoglycans.^72^^,^^73^ When applied to scarred corneas, and because it targets the principal causes leading to burn-induced LSCD and vision loss, BMT represents a potential route to eliciting true tissue regeneration as a therapeutic outcome. This novel treatment relies on technically simple, highly accessible, and inexpensive methods, thus providing a pharmacological alternative for restoring the normal mechanical properties of stiffened tissues without the need for surgical intervention or stem cell transplant (Fig. 1).

BMT acts by promoting maintenance and proliferation of endogenous LESCs, accelerating re-epithelization, and helping burn tissues to heal even when used topically (Fig. 2i). This advantage obviates the need to remove the epithelium prior to treatment, thus preventing long-term clinical complications associated with corneal debridement and secondary proinflammatory response. BMT may also be proven useful as an adjunct to LESC transplantation in clinical cases with total LSCD prognosis or diagnosis (Fig. 2ii). A damaged limbal microenvironment can limit the use of therapeutic procedures, with newly transplanted LESCs being negatively affected by compromised (i.e., stiffened) substrates in the post-injury cornea. BMT can thus be indicated to soften the host stroma with minimal risks of post-operative complications due to inflammation (Fig. 1). Conversely, BMT can be used to better design the appropriate mechanical properties in natural or manufactured carriers for LESC transplantation or the engineering of tissue constructs. Indeed, the prevalent use of substrates with limbus-like compliance (e.g., fibrin gels, hAM)^74^^,^^75^ for the ex vivo expansion of LESCs is, however accidental, probably incidental to the success of the subsequent transplantation. Ultimately, the use of BMT to control the mechanical properties of tissues in injured corneas may represent, alone or in combination, the most sophisticated, single-use, non-intrusive strategy for achieving corneal repair based on the long-lasting restoration of normal cellular healing responses (Fig. 2).

## Future Perspectives

This concise review has analyzed how recent fundamental discoveries in corneal and stem cell mechanobiology have contributed to the development of BMT, a new pharmacological approach for the treatment of tissue-stiffening pathologies. Evidence that tissue-softening enzymes such as collagenase type I can be safely used to treat corneal burns may appear, at least under cursory analysis, to be nonsensical considering the deleterious role endogenous proteases have in the post-burn corneal response.^11^ This is not the case, however, as BMT relies on the use of very specific enzyme types that cleave only fibrillar collagen (instead of broad-spectrum proteases) and that are applied only once, at low doses (i.e., 50–200 µg·mL^–^^1^, orders of magnitude below the 5–10 mg·mL^–^^1^ necessary to compromise tissue integrity^76^^,^^77^), and for durations compatible with clinical interventions (15 minutes or less, instead of persistently).^52^ Nevertheless, and despite its promising attributes, further studies will be necessary before BMT can be used in a clinical ophthalmology setting. These will involve first-in-human clinical trials for the adjunct treatment of LSCD patients (e.g., caused by chemical burns) and the use of medical-grade collagenase in longer term experiments to determine how enduring the beneficial effects are and to monitor for late adverse effects. In addition, testing in LSCD corneas ex vivo will allow us to ascertain if increased stiffness varies with injury severity and to determine if individual protocol adjustments are required. Finally, we believe that future studies aimed at developing alternative methods for modulating tissue biomechanics or controlling stem cell phenotype via mechanotransduction signaling (e.g., using biochemical or molecular biology tools to regulate BMP4 expression)^58^ merit further consideration and can find extended applications in ophthalmology and beyond.^78^
